# Whole Genome Sequencing and Comparative Genome Analyses of *Chlamydia abortus* Strains of Avian Origin Suggests That *Chlamydia abortus* Species Should Be Expanded to Include Avian and Mammalian Subgroups

**DOI:** 10.3390/pathogens10111405

**Published:** 2021-10-29

**Authors:** Kinga Zaręba-Marchewka, Monika Szymańska-Czerwińska, Morag Livingstone, David Longbottom, Krzysztof Niemczuk

**Affiliations:** 1 Department of Cattle and Sheep Diseases, National Veterinary Research Institute, Al. Partyzantow 57, 24-100 Pulawy, Poland; monika.szymanska@piwet.pulawy.pl (M.S.-C.); kniem@piwet.pulawy.pl (K.N.); 2 Laboratory of Serological Diagnosis, National Veterinary Research Institute, Al. Partyzantow 57, 24-100 Pulawy, Poland; 3 Moredun Research Institute, Pentlands Science Park, Bush Loan, Penicuik, Midlothian EH26 0PZ, UK; morag.livingstone@moredun.ac.uk (M.L.); david.longbottom@moredun.ac.uk (D.L.)

**Keywords:** *Chlamydia*, avian *C. abortus*, WGS, Illumina, Nanopore

## Abstract

A variety of *Chlamydia* species belonging to the *Chlamydiaceae* family have been reported in birds. Until recently, *C. psittaci* was considered to be the most common avian species, although found in both birds and mammals, while *C. abortus* has only been found in mammals. Recently, a new group of avian *C. abortus* strains with worldwide distribution in various wild bird families has been described. In this study, whole genome sequencing (WGS) of three of these strains (15-70d24, 15-49d3 and 15-58d44, representing genotypes G1, G2 and 1V, respectively) that were isolated from wild birds were analysed. Genome assemblies based on both short-read Illumina and long-read Nanopore data indicate that these avian *C. abortus* strains show features characteristic of both *C. abortus* and *C. psittaci* species, although phylogenetic analyses demonstrate a closer relationship with classical *C. abortus* strains. Currently, species classification established by the ICSP Subcommittee on the taxonomy of Chlamydiae, determines that these avian *C. abortus* strains 15-70d24, 15-49d3 and 15-58d44 should be classified as *C. abortus*. However, the authors of this study conclude that the current taxonomic definition of *C. abortus* is outdated and should be amended to include two subgroups, mammalian and avian, the latter of which would include all isolates so far referred to as atypical *C. psittaci* or *C. psittaci*/*C. abortus* intermediates.

## 1. Introduction

Birds as well as other animals can be carriers of various pathogens, including the obligate intracellular bacteria of the family *Chlamydiaceae*. This family currently consists of the two genera: *Chlamydia* (*C.*) and recently described *Chlamydiifrater* [1]. Genus *Chlamydia* includes 14 recognised species: *C. abortus*, *C. avium*, *C. buteonis*, *C. caviae*, *C. felis*, *C. gallinacea*, *C. muridarum*, *C. pecorum*, *C. pneumoniae*, *C. poikilothermis*, *C. psittaci*, *C. serpentis*, *C. suis*, and *C. trachomatis* [2,3,4,5]. Moreover, there are four *Candidatus* species *(Cand.)*: *Cand.* C. ibidis, *Cand.* C. sanzinia, *Cand.* C. corallus and *Cand*. C. testudinis [6,7,8,9]. Chlamydiae are widely distributed throughout the world causing a variety of diseases both in humans and animals, including zoonoses [10,11]. *C. psittaci*, which is encountered mainly in birds, has been considered for a long time as the sole species causing avian chlamydiosis [12,13,14]. However, it has been shown that other chlamydial species can also be harboured by birds. In addition to the classical *C. abortus*, *C. pecorum*, *C. trachomatis*, *C. suis* or *C. muridarum* species [15,16,17], new chlamydial species have been detected in birds within recent years: *C. avium* isolated from pigeons and psittacine birds, *C. gallinacea* carried by domestic poultry, *C. buteonis* in raptors and *Cand.* C. ibidis in the African sacred ibis [5,6,18]. Scientific reports show that the spectrum of chlamydial lineages occurring in birds is wide. Latterly, a new group of avian *C. abortus* strains with worldwide distribution in various wild bird families (Anatidae, Corvidae, Psittacidae, Rallidae) were described [19,20]. These non-classified chlamydial strains were previously referred as atypical *C. psittaci* or *C. psittaci*/*C. abortus* intermediates as they were demonstrating features of both *C. psittaci* and *C. abortus*. Most recently, the genome sequence of one of these strains, *C. psittaci* strain 84/2334 has been described and the strain reclassified as *C. abortus* species [21]. Availability of whole genome sequencing (WGS) provides a unique opportunity in understanding the genetic diversity and biology of the novel taxa in the genus *Chlamydia*, and hence to create a taxonomic definition. Recently, we have announced partial draft genome sequences of three avian *Chlamydia abortus* strains corresponding to genotypes G1, G2 and 1V (strains 15-70d24, 15-49d3 and 15-58d44, respectively), isolated from wild birds in Poland, based on Illumina sequencing [22,23]. As the genomes were incomplete, we decided to improve sequence data and perform confirmatory resequencing based on short-read Illumina and long-read Nanopore Technology to facilitate contig gap closure and conduct in-depth genome analysis. Here, we describe the results of this hybrid sequence analysis and phenotypic features of these three avian *C. abortus* strains.

## 2. Results 

### 2.1. Genomes of Avian C. abortus (Strains 15-70d24, 15-49d3 and 15-58d44, Representing Genotypes G1, G2, 1V, Respectively) Based on Hybrid Sequencing

Sequencing resulted in single circular chromosomal contigs for each of the strains 15-70d24, 15-49d3, 15-58d44, with a length of 1,141,680, 1,132,330 and 1,151,406 bp, and average sequence coverage of 468×, 2437× and 7427×, respectively. G+C content for all three strains at 39.6–39.9% was more in keeping with that of classical *C. abortus* (39.9%; [21]) than *C. psittaci* (38.8–39.1%; [21]) strains. Single contig plasmid sequences for 15-70d24, 15-49d3 and 15-58d44 with lengths of 7553, 7556 and 7553 bp and sequence coverage of 1692×, 8017× and 9043×, respectively, were also extracted from the total whole genome raw sequence reads by comparison with other *C. psittaci* plasmid sequences. Detailed properties of the genomes in comparison to representatives of the family *Chlamydiaceae* are presented in Supplementary Table S1. The genome sequences of avian *C. abortus* (strains 15-70d24, 15-49d3 and 15-58d44) chromosomes and plasmids based on hybrid sequencing and Nanopore raw data obtained in this study have been deposited in European Nucleotide Archive (ENA) under the following accession numbers: LS450958.2, LS450956.2, OU508367.1 (chromosomes); LS450959.2, LS450957.2, OU508368.1 (plasmids); ERR6415086, ERR6415087, ERR6415088 (Nanopore), respectively. Accession numbers for Illumina raw data have been published previously [22,23]. All accession numbers related to WGS of avian *C. abortus* strains 15-70d24, 15-49d3 and 15-58d44 (G1, G2 and 1V, respectively) are included as a part of bioproject PRJEB26715.

### 2.2. Phenotypic Characterisation of Avian C. abortus Strains Corresponding to Genotypes G1, G2 and 1V (Strains 15-70d24, 15-49d3 and 15-58d44)

**Figure 1:**
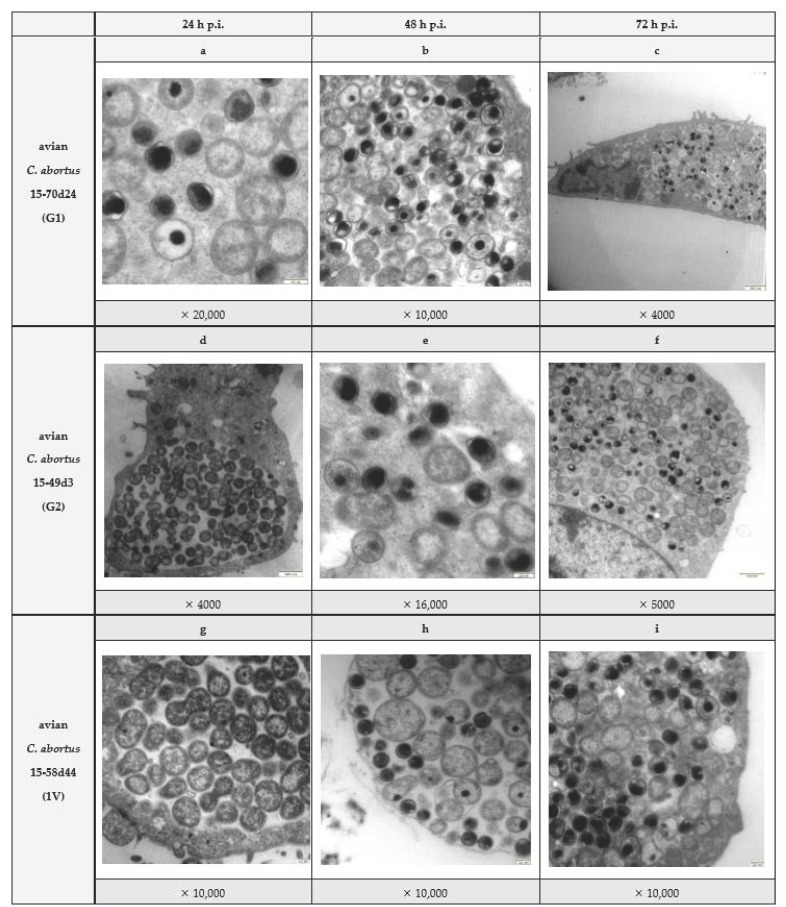
Transmission electron microscopy images of Buffalo Green Monkey (BGM) cell culture infected with avian *C. abortus* strains. Top row (images (**a**–**c**)) presents BGM cell culture infected with avian *C. abortus* 15-70d24 after 24, 48 and 72 h, respectively. Second row (images (**d**–**f**)) shows BGM cell culture infected with avian *C. abortus* 15-49d3 after 24, 48 and 72 h, respectively. Bottom row (images (**g**–**i**)) illustrates BGM cell culture infected with avian *C. abortus* 15-58d44 after 24, 48 and 72 h, respectively. The magnification is given below images.

Strains were isolated and propagated according to routine established standard procedures in Buffalo Green Monkey (BGM) cells [19,22,23]. By transmission electron microscopy (TEM), the typical biphasic developmental cycle was observed as seen for all chlamydiae. Figure 1 shows inclusions in infected BGM cells including elementary bodies (EBs), reticulate bodies (RBs), dividing reticulate bodies (dividing RBs) and intermediate bodies (IBs). In all three isolates, EBs are round, electron dense and 240–400 nm in diameter. RBs are larger (400–1100 nm), round to oval and more electron lucent. Binary fission of RBs was observed at different stages of division. Occasionally, IBs with characteristic electron dense centres and a more lucent periphery were found. Immunofluorescence testing of all strains displayed positive reaction with a monoclonal antibody to chlamydial LPS (IMAGEN Chlamydia Kit, Oxoid, Wesel, Germany) (not shown).

### 2.3. Genome Analysis

#### 2.3.1. 16S rRNA and 23S rRNA Phylogenetic Analyses

**Figure 2:**
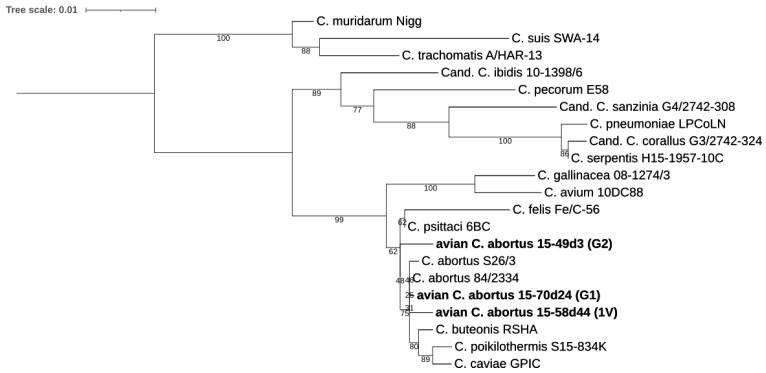
Analysis of the complete 16S rRNA sequences of the avian *C. abortus* strains 15-70d24, 15-49d3, 15-58d44 (G1, G2, 1V, respectively) and representative strains of *Chlamydia* spp. Phylogeny based on 1567 bp consensus alignment was constructed by Maximum Likelihood method with best-fit model according to Bayesian Information Criterion: TVM+F+I+G4. Bootstrap values are presented as percentages.

**Figure 3:**
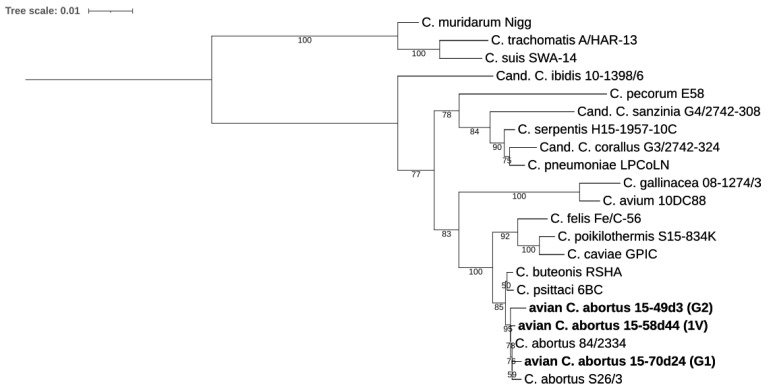
Analysis of the complete 23S rRNA sequences of the strains 15-70d24, 15-49d3 and 15-58d44, representing genotypes G1, G2 and 1V, respectively, and members of *Chlamydiaceae*. Phylogeny based on 2943 bp consensus alignment was constructed by Maximum Likelihood method with best-fit model according to Bayesian Information Criterion: TPM3u+F+I+G4. Bootstrap values are presented as percentages.

The taxonomic position of the avian *C. abortus* strains within the genus *Chlamydia* was evaluated based on pairwise sequence identities, with values greater than 92.5% and 91% for 16S rRNA (Figure 2) and 23S rRNA genes (Figure 3), respectively, identified [24]. Sequence homologies showed their closest relatedness was to *C. abortus*, *C. psittaci* and *C. buteonis*. Pairwise sequence identity values of representative strains for *Chlamydiaceae* and the three avian *C. abortus* strains 15-70d24, 15-49d3 and 15-58d44 are presented in Supplementary Table S2.

#### 2.3.2. Molecular Typing of Avian *C. abortus* Strains

**Figure 4:**
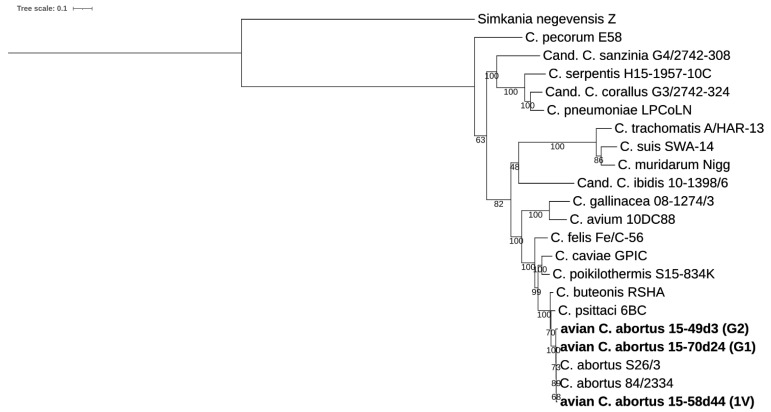
Analysis of the nine phylogenetically informative markers. The dendrogram was constructed by Maximum Likelihood method with best-fit model according to Bayesian Information Criterion: JTT+F+G4 model based on the concatenated alignment of 9 individual genes (*DnaA, SucA, Hyp325, Fabl, RpoN, FtsK, PepF, Adk, HemL*), and includes avian *C. abortus* strains 15-70d24, 15-49d3, 15-58d44 (G1, G2, 1V, respectively), and representative strains of the genus *Chlamydia*. Bootstrap values are presented as percentages. The tree scale bar indicates the number of amino acid substitutions per site.

The identity values obtained for the strains 15-70d24, 15-49d3, 15-58d44 representing genotypes G1, G2 and 1V, respectively, are above the accepted cut-off for a new species assignment, so the isolates can be assigned to the *Chlamydia* genus (DnaA  ≥  70%, Fabl  ≥  78%, Hyp325  ≥  57% and SucA  ≥  64%) and strains of *C. abortus* (RpoN  >  96%, PepF  >  96%, Adk  >  95%, FtsK  >  98%, HemL  >  95%) [24]. Detailed results are shown in Figure 4 and Supplementary Tables S3–S5.

#### 2.3.3. ANIb and Tetra-Nucleotide Signatures

Average nucleotide identity by BLAST (ANIb) and tetranucleotide regressions were calculated based on avian *C. abortus* strains (15-70d24, 15-49d3 and 15-58d44, representing genotypes G1, G2 and 1V, respectively) in comparison to genomic sequences of *Chlamydiaceae* representatives. The results were presented in Supplementary Table S6. Highest values of 0.99881, 0.99783, 0.9989 (tetranucleotide regression) and 97.93, 97.47, 98.36 (ANIb) were noted between 15-70d24, 15-49d3, 15-58d44, respectively, and UK reference strain *C. abortus* S26/3.

#### 2.3.4. Plasmid Comparisons 

**Figure 5:**
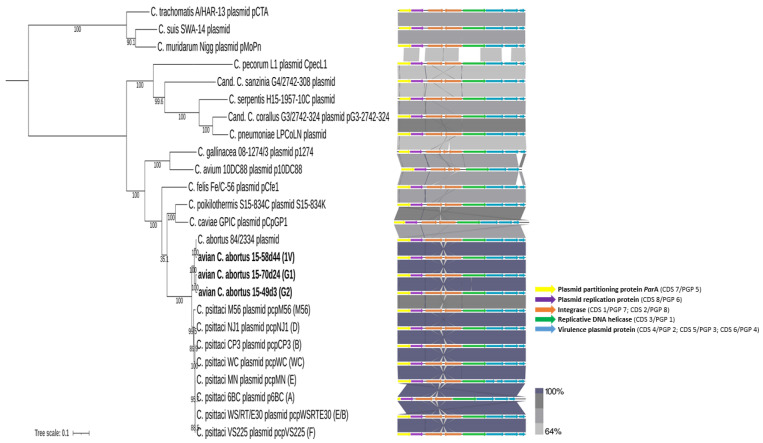
Phylogenetic analysis of avian *Chlamydia abortus* strains corresponding to genotypes G1, G2 and 1V (strains 15-70d24, 15-49d3 and 15-58d44, respectively) and *Chlamydia* spp. based on plasmid sequences comparison and chlamydial plasmids arrangement. Support values are presented on the branches. Figure was constructed using EasyFig. Grey shading represents tBLASTx matches, while coloured arrows represent coding regions.

Plasmids were detected in all three avian *C. abortus* genotypes. They are organised into eight open reading frames (ORFs) (Figure 5), as observed for all other chlamydial plasmids. Avian *C. abortus* 15-70d24, 15-49d3, 15-58d44 and *C. abortus* 84/2334 show the greatest sequence similarity: 97.63%, 97.33% and 99.54%, respectively. *C. psittaci* plasmids have significant sequence similarity, ranging from 94.48% to 95.75% nucleotide identity depending on genotype (Figure 5; Supplementary Table S7). *C. psittaci* 6BC (genotype A) shows the lowest nucleotide identity compared to 15-70d24, 15-49d3, 15-58d44 plasmids (94.61%, 94.48%, 94.68%, respectively). The highest level of nucleotide identity among *C. psittaci* is observed between avian *C. abortus* 15-70d24 and *C. psittaci* CP3 and *C. psittaci* NJ1, representing genotypes B and D, respectively (95.71%); avian *C. abortus* 15-49d3 and *C. psittaci* WS/RT/E30 and *C. psittaci* VS225 representing genotypes E/B and F, respectively (95.47%); and avian *C. abortus* 15-58d44 and *C. psittaci* CP3 being representative of genotype B (95.75%). 

#### 2.3.5. Plasticity Zone

**Figure 6:**
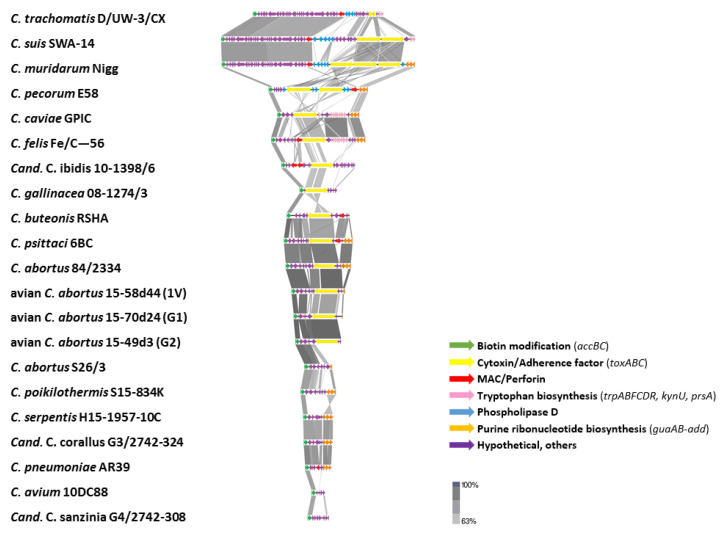
Graphical presentation of plasticity zones of avian *Chlamydia abortus* strains 15-70d24, 15-49d3, 15-58d44 representing genotypes G1, G2, 1V, respectively, and representatives of *Chlamydia* spp. and *Candidatus* to this species via tBLASTx analysis and their arrangement plotted in EasyFig. The coloured arrows represent genes of plasticity zones according to their function, while grey shading represents sequence homology (as listed in the legend).

The plasticity zone (PZ) region (19,443-22,657 bp) of avian *C. abortus* strains 15-70d24, 15-49d3, 15-58d44 (G1, G2, 1V, respectively) is composed of genes required for several biochemical pathways such as acetyl-CoA-carboxylase (*acc*BC) and partial purine and pyrimidine biosynthesis genes (*gua*AB-add). When compared with other chlamydial species, PZs of avian *C. abortus* isolates are most related to the *C. abortus* 84/2334 (51.50–77.67%), *C. buteonis* RSHA (54.47–70.85%)*, C. psittaci* 6BC (49.12–64.19%), *Cand.* C. ibidis 10-1398/6 (39.67–43.08%), *C. abortus* S26/2 (25.73–38.38%) and *C. gallinacea* 08-1274/3 (25.61–26.24%) (Figure 6, Supplementary Table S8). In contrast to classical *C. abortus* S26/3 strain, the avian *C. abortus* 15-70d24, 15-49d3 and 15-58d44 strains contain the cytotoxin virulence factor in their genomes, which is characteristic for *C. abortus* 84/2334, *C. psittaci* 6BC and *C. buteonis* RSHA strains. On the other hand, *C. psittaci* 6BC and *C. buteonis* RSHA strains have MAC/perforin genes which are absent in avian *Chlamydia abortus* genomes and classical *C. abortus* strains. Additionally, avian and typical *C. abortus* representatives have a smaller PZ than most of chlamydial species. No tryptophan operon (*trpABFCDR*, *kynU*, *prsA*) and phospholipase D were detected in the plasticity zone of avian *C. abortus* strains.

#### 2.3.6. Phylogenetic Network Analysis

**Figure 7:**
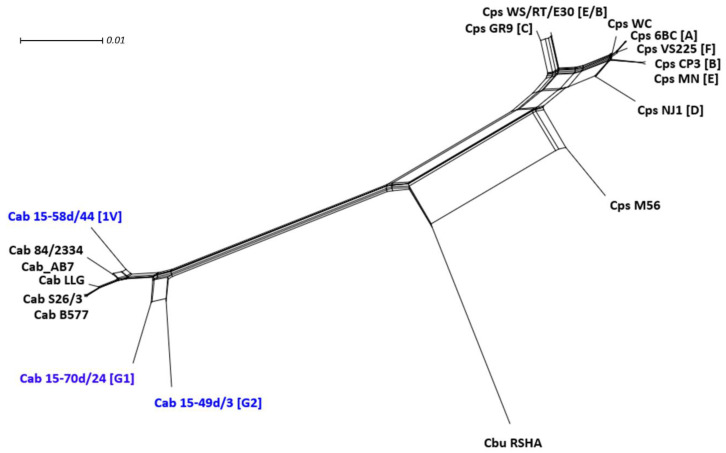
NeighborNet tree of *C. abortus*, *C. psittaci* (genotypes A, B, C, D, E, E/B, F, M56 and WC), *C. buteonis* and avian *C. abortus* (genotypes G1, G2 and 1V) representatives. The scale bar indicates the number of substitutions per site.

The results show that the avian *C. abortus* G1, G2 and 1V strains are placed together with *C. abortus* representatives. *C. psittaci* strains create a separate clade, while *C. buteonis* is positioned between *C. abortus* and *C. psittaci* representatives (Figure 7).

#### 2.3.7. SNP Analysis

**Figure 8:**
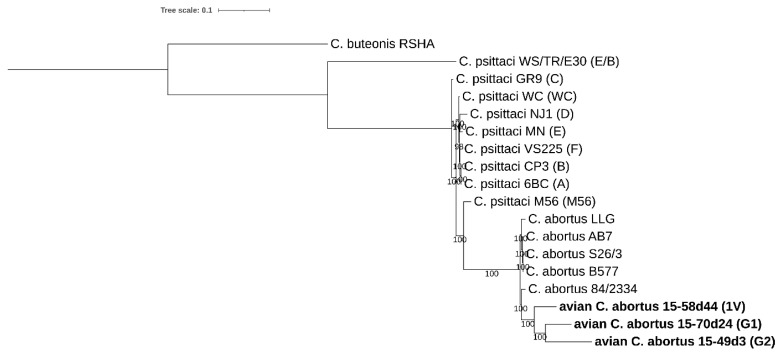
Single-nucleotide polymorphism-based phylogeny of *C. abortus*, *C. psittaci* (genotypes A, B, C, D, E, E/B, F, M56 and WC), *C. buteonis* and avian *C. abortus* (genotypes G1, G2 and 1V) strains. The dendrogram was constructed by Maximum Likelihood method with best-fit model according to Bayesian Information Criterion: K3P. Bootstrap values are presented as percentages. The tree was rooted with *C. buteonis* as an outgroup. The scale bar indicates the number of substitutions per site.

Whole-genome single-nucleotide polymorphism (SNP) analysis clearly clustered all strains into three groups: *C. buteonis*, *C. psittaci* and *C. abortus* (Figure 8). Avian *C. abortus* strains 15-70d24, 15-49d3, 15-58d44 are grouped together with *C. abortus* strains but create a subclade. The number of SNP differences for 15-70d24, 15-49d3, 15-58d44 within subclade is ranging from 13,071 to 16,891, while within *C. abortus* clade is ranging from 8872 to 16,730. Interestingly, the number of SNP differences between 15-58d44 and classical *C. abortus* strains and *C. abortus* 84/2334 is remarkably lower (8872–9554) than for 15-70d24 (11,957–12,637) or 15-49d3 (16,064–16,730). Significantly greater number of SNP differences is observed between avian *C. abortus* strains and *C. psittaci* representatives (21,854–69,925) and *C. buteonis* (72,206–74,315) (Supplementary Table S9).

## 3. Discussion

Molecular analysis of avian *C. abortus* performed in our previous study [19] did not lead to final determination of their taxonomic position because the isolates demonstrated intermediate features of both *C. abortus* and *C. psittaci*. Previous research has suggested that these three strains should be classified as avian *C. abortus* strains based on preliminary analysis. Although, MLST analysis indicated they should be classified as *C. abortus*, it was not clear if they should be considered as a part of the established classical *C. abortus* species or be designated a completely new species. Therefore, complete information based on WGS analysis was required. In this study, a more detailed molecular analysis of the genome sequences was performed to fully characterize these strains. In the last decade, whole-genome sequence analysis has enabled identification and characterization of new taxa: *C. avium*, *C. gallinacea* [18,25], *C. buteonis* [5], *C. poikilothermis* [3], *C. serpentis* [3], *Cand.* C. ibidis [6], *Cand.* C. sanzinia [7], *Cand.* C. corallus [8] and *Cand.* C. testudinis [9]. Genome sequencing based on short-read technologies is relatively inexpensive and widely accessible, but due to repetitive regions and/or high level of %G+C content it can be challenging to close the genome. Long-read sequencing however can span problematic repetitive regions thus improving assembly success. On the other hand, long-read sequencing has several drawbacks including higher error rates affecting both insertions and deletions [26] but it should be noted that error rates have significantly come down to nearer 5% using the latest Nanopore sequencing technologies. In this study, a hybrid sequencing approach, combining the high-throughput (Oxford Nanopore Technologies) and high-accuracy short read data (Illumina), was utilized to produce single contiguous genome sequences of avian *C. abortus* strains 15-70d24, 15-49d3 and 15-58d44 corresponding to genotypes G1, G2 and 1V, respectively [22,23]. This approach provides reliable contigs and scaffolds by firstly assembling short-read data to produce an assembly graph and then using long-reads to create bridges between contigs and scaffolds. High-quality hybrid assembly can help resolve difficulties associated with repeat regions and with the identification of SNPs. Therefore, hybrid sequencing seems to be the gold-standard in genomic analysis [27].

Analysis of the three strains, 15-70d24, 15-49d3 and 15-58d44, originating from *Anas crecca, Anas platyrhynchos* and *Pica pica*, respectively, revealed that they belong to the order Chlamydiales (identity values of ≥80% for 16S rRNA and 23S rRNA) and family *Chlamydiaceae* (identity values of ≥92.5% for 16S rRNA, ≥91% for 23S rRNA) based on the classification scheme proposed by Pillonel et al [24]. According to the comparison of nine informative marker proteins with 15 members of the *Chlamydia* genus and three *Candidatus* species, the new isolates could be assigned to species *C. abortus* (RpoN  >  96%, PepF  >  96%, Adk  >  95%, FtsK  >  98%, HemL  >  95%) of the *Chlamydia* genus (DnaA  ≥  70%, Fabl  ≥  78%, Hyp325  ≥  57% and SucA  ≥  64%) [24]. G+C content of G1, G2 and 1V (39.9%, 39.6%, 39.8%) was mostly similar to *C. abortus* 84/2334 (39.9%) and classical *C. abortus* S26/3 strain (39.9%). The ANIb and tetra nucleotide regression values calculated for 15-70d24, 15-49d3 and 15-58d44 also indicated they should be classified as *C. abortus* species. 

Interestingly, comparative genome analysis revealed that the G1, G2 and 1V strains significantly differ from classical *C. abortus* strains. Comparative genomics of *Chlamydia* spp. is mainly focused on the plasticity zone (also known as the replication termination region–RTR) as a key region of chlamydial genomes associated with pathogenesis [28,29,30]. The size of plasticity zones in chlamydial genomes ranges usually from 6 kb to 83 kb [3,5,6,7,8,9,28,29,30] dependent on species and is bounded by acetyl-CoA carboxylase (*accB*) and einosine-5′-monophosphate dehydrogenase (*guaB*) [28,29]. The lack or truncation of the *guaAB-add* operon is common for some species including *C. trachomatis*, *C. suis*, *C. gallinacea*, *C. avium*, *C. buteonis* and avian *C. abortus*. This highly variable region contains genes encoding proteins responsible for regulation of fatty acid synthesis and degradation (*accBC*), purine biosynthesis (*guaAB-add*) and tryptophan biosynthesis (*trpABFCDR, kynU, prsA*). Moreover, it comprises several virulence factors including cytotoxin genes/adherence factor, membrane attack complex/perforin (MAC/Perforin) and phospholipase D (PLD) enzymes as well as a number of hypothetical proteins of unknown function [28,29]. Avian *C. abortus* strains have a more extensive PZ when compared to the classical *C. abortus* S26/3 representative. Considering the size, PZs of avian *C. abortus* 15-70d24 (20,909 bp), 15-49d3 (19,443 bp) and 15-58d44 (22,657 bp) are most similar to *C. abortus* 84/2334 (27,678 bp), *C. psittaci* 6BC (29,144 bp) and *C. buteonis* RSHA (26,735 bp), while PZ of *C. abortus* S26/3 is about half the size (11,776 bp). Analysis of nucleotide identity of avian *C. abortus* strains showed their structure is most similar to: *C. abortus* 84/2334 (51.50–77.67%), *C. buteonis* RSHA (54.47–70.85%) and *C. psittaci* 6BC (49.12–64.19%). Moreover, the avian *C. abortus* strains in this study carry the cytotoxin gene which is absent in classical *C. abortus* S26/3 but present in *C. abortus* 84/2334, which is also of avian origin [21]. However, in contrast to *C. psittaci* 6BC and *C. buteonis* RSHA, the MAC/Perforin gene is absent from the avian *C. abortus* PZ, as previously observed for *C. abortus* S26/3 and *C. abortus* 84/2334 [21,31]. It has been suggested that MAC/Perforin might be involved in assisting PLD proteins in lipid acquisition and processing [29] but no PLDs were detected in avian *C. abortus* strains neither were they detected in *C. abortus* S26/3, *C. psittaci* 6BC or *C. buteonis* RSHA, or observed in the recently published avian *C. abortus* 84/2334 strain [21]. The complete *trp* operon is absent in avian *C. abortus* which is a characteristic feature of the majority of *Chlamydia* spp. excluding *C. caviae* and *C. felis.* It probably suggests a different pathway for synthesising tryptophan.

Most chlamydial species, but not all strains, carry an endogenous 7.5 kbp circular plasmid which has been identified in *C. avium* [18], *C. caviae* [32], *C. felis* [33], *C. gallinacea* [18], *C. muridarum* [34], *C. pecorum* [35], *C. pneumoniae* [36], *C. poikilothermis* [3], *C. psittaci* [37,38], *C. serpentis* [3], *C. suis* [39], *C*. *trachomatis* [2], *C. buteonis* [5], *Cand.* C. sanzinia [7], *Cand.* C. corallus [8] and *Cand.* C. testudinis [9]. It is assumed that the presence of plasmid is associated with the virulence of the strain. Plasmids are highly conserved and not integrated into the genome [36,40,41]. They usually contain eight coding sequences (CDS) encoding for genes involved in plasmid maintenance and glycogen synthesis [42,43] and non-coding RNA with unknown functionality [41]. Microbial plasmids can carry antibiotic resistance genes, but there is no proof of antibiotic resistance located on chlamydial plasmids [44]. Interestingly, all of the avian C. *abortus* strains reported here possess plasmids, similar to that found in the avian *C. abortus* strain 84/2334, while to date no plasmids have been identified in classical *C. abortus* strains [21]. The size of the avian *C. abortus* plasmids ranging from 7553 to 7556 bp and their sequences are most closely related to *C. abortus* 84/2334 (97.33–99.54%) and *C. psittaci* strains ranging from 94.48% to 95.75%. It should be highlighted that avian *C. abortus* (strains 15-70d24, 15-49d3 and 15-58d44) plasmids share the most similar structure with the plasmids of *C. abortus* 84/2334, isolated from a parrot, and genotypes B, D, E/B and F of *C. psittaci*, which were isolated from pigeon, turkey, mallard and parakeet, respectively, whereas *C. psittaci* genotype A strain associated with parrot shares the lowest nucleotide identity with plasmids of avian *C. abortus*. Plasmid proteins are commonly used for diagnostic targets and vaccine candidates, therefore future research on avian *C. abortus* plasmids might lead to the development of new vaccines against avian *C. abortus* and/or *C. psittaci* strains [45].

Whole-genome SNP and NeighborNet analyses clearly show that the avian *C. abortus* strains 15-70d24, 15-49d3 and 15-58d44 are phylogenetically positioned as *C. abortus* species confirming that they should be classified as *C. abortus*. Interestingly, the number of SNP differences indicate that the strain 15-58d44 is phylogenetically more closely related to classical *C. abortus* strains and *C. abortus* 84/2334 (8872–9554) than to the 15-70d24 (13,071) and 15-49d3 (16,891) strains.

Avian *C. abortus* strains can be found in birds, mainly in waterfowl (genotype G1 and G2) and in corvids (genotype 1V). The microorganism can be isolated from cloaca and oropharynx. The route of transmission has not yet been determined but by analogy with other Chlamydiae, infection may take place through airborne aerosols containing faecal and dust particles or direct contact with fresh faeces. Zoonotic potential of these strains remains unknown but may become clearer in time as more strains are identified in human populations. Due to the fact that more than 50% of avian *C. abortus* DNA extracts are non-typable by the available PCR assays [19], our team is currently working on development of a specific real-time PCR assay. 

Taking into consideration the above, the plasmid and chromosomal genome characteristics and WGS comparison indicated that avian *C. abortus* show features of both *C. abortus* and *C. psittaci* species, in agreement with analyses recently published on avian *C. abortus* strain 84/2334 [21]. However, based on species classification rules established by the International Committee on Systematics of Prokaryotes Subcommittee on the taxonomy of Chlamydiae, they should be classified as *C. abortus*. Furthermore, as previously suggested, current definition of *C. abortus* is outdated and should be expanded to include not only the classical mammalian isolates, but also strains isolated from birds 15-70d24, 15-49d3, 15-58d44 (G1, G2, 1V, respectively), Prk/Daruma and 84/2334 [4,21,46,47,48]. 

## 4. Materials and Methods

### 4.1. Bacterial Strains

During the previously published scientific work [19,22,23], cloacal/faecal swabs were taken from birds transiently living in bird rehabilitation centres and free-living birds caught randomly by authorised veterinarians or ornithologists during clinical studies. According to the Local Ethical Committee on Animal Testing at the University of Life Sciences in Lublin (Poland), formal ethical approval is not required for this kind of study [19]. The individual health status of a bird was not recorded. Avian *C. abortus* strains representing genotype G1 (15-70d24) from Eurasian teal *(Anas crecca)*, genotype G2 (15-49d3) from mallard *(Anas platyrhynchos)* and 1V (15-58d44) from magpie *(Pica pica)* were successfully isolated in BGM cell culture and propagated in 25 cm^2^ tissue culture flasks for 72 h based on procedures published previously [19,22,23]. To confirm proper multiplication of the strains, a *Chlamydiaceae*-specific real-time PCR targeting the 23S rRNA gene fragment [49] and immunofluorescence staining using IMAGEN *Chlamydia* kit (Oxoid, Wesel, Germany), according to manufacturer’s instructions, were performed. Deep molecular analysis of *omp*A, 16S rRNA, IGS, partial 23S rRNA sequences and MLST as well as announcements of the draft genomes were described in our previous studies [19,22,23]. Basic data of the cultured isolates presented in this work are specified in Supplementary Table S10. Strains used in this study and their GenBank accession numbers are listed in Supplementary Table S11.

### 4.2. Electron Microscopy

BGM cells were infected with avian *C. abortus* strains corresponding to genotypes G1, G2 and 1V (strains 15-70d24, 15-49d3 and 15-58d44, respectively). At 24, 48 and 72 h post-infection, infected cells plus medium were harvested by scraping from the flasks and transferred to separate micro-centrifuge tubes before adding 4% glutaraldehyde solution to each tube at a 1:1 ratio. Tubes were centrifuged at 1000× *g* for 10 min and pellets were used for preparing microscope slides. *Chlamydia*-infected cells were fixed in 2% paraformaldehyde/2.5% glutaraldehyde (Roth, Karlsruhe, Germany) in 100 mM phosphate buffer, pH 7.2, for 1 h at room temperature. Cells were washed in phosphate buffer and post-fixed in 1% osmium tetroxide (Roth, Karlsruhe, Germany) for 1 h. After several rinses in distilled water, samples were dehydrated in a graded series of ethanol and embedded in Epon 812 resin (Sigma Aldrich, Saint Louis, MO, USA). Sections of 70–80 nm were cut, stained with uranyl acetate and lead citrate, put on grids and viewed on a Zeiss Libra 120 transmission electron microscope (Carl Zeiss, Oberkochen, Germany). Evaluation of phenotypic features was performed including diameter of elementary/reticulate bodies and confirmation of biphasic developmental cycle.

### 4.3. Genomic DNA Preparation

#### 4.3.1. Illumina Sequencing

Genomic DNA was extracted after several passages from BGM cell culture infected with avian *C. abortus* strains 15-70d24, 15-49d3 and 15-58d44 by DNeasy Blood and Tissue Kit (Qiagen, Hilden, Germany) following the manufacturer’s instructions. Removal of methylated host DNA from obtained genomic DNA samples was performed according to the manufacturer’s protocol of NebNext Microbiome DNA Enrichment Kit (BioLabs, Ipswich, UK) [22,23].

#### 4.3.2. Nanopore (MinION) Sequencing

Infected cells from ten 225 cm^2^ infected tissue culture flasks were harvested using sterile glass beads and centrifuged at 153× *g* for 10 min at 4 °C to remove gross cellular debris. Supernatants were then centrifuged at 22,100× *g* for 30 min at 4 °C and pellets were washed with ice-cold PBS and centrifuged as previously. Each pellet was resuspended in 20 mM Tris-HCl, pH 7.5/150 mM KCl/1% sarkosyl, lightly homogenised using a ground glass homogenizer and carefully layered onto 3 mL cushions of 15% sucrose in 20 mM Tris-HCl, pH 7.5/150 mM KCl/1% sarkosyl before centrifuging at 70,000× *g* for 45 min at 4 °C. Genomic DNA was extracted from pellets using DNeasy Blood and Tissue Kit (Qiagen, Hilden, Germany) as per manufacturer’s instructions.

#### 4.3.3. Quality Assessment and Quantification

To confirm the quality and quantity of extracted DNA, spectrophotometric and fluorometric methods were used. DNA extracts were checked qualitatively and quantitatively by means of Nanodrop spectrophotometer (DeNovix, Wilmington, NC, USA) and Qubit 3.0 fluorometer (Thermo Fisher Scientific, Waltham, MA, USA).

### 4.4. Genome Sequencing, Assembly and Draft Annotation

#### Illumina and Nanopore Sequencing

Genomic DNA libraries were prepared using the Nextera XT DNA library preparation kit and Nextera XT index kit (Illumina, San Diego, CA, USA). Sequencing was carried out on MiSeq sequencer (Illumina, San Diego, CA, USA) with the 2 × 300-bp paired-end protocol [22,23]. Long-read genomic DNA libraries were prepared using a rapid sequencing kit (SQK-RAD004; Oxford Nanopore Technologies [ONT], Oxford, UK). Sequencing was conducted in a MinION using a FLO-MIN106D (R9) flow cell according to a standard protocol (ONT, Oxford, UK) and basecalled with Guppy v.3.0.7. Illumina raw sequencing data were processed using fastp v.0.20.1 [50] for the purpose of trimming adapters and low-quality data. Long reads were adapter trimmed with Porechop v.0.2.4. BBDuk v.38.34 [51] was applied to remove non-chlamydial reads pertaining to the host DNA (from BGM cells) based on standard operating procedures. *De novo* genome assembly based on both the Illumina and Nanopore (MinION) data was performed using the hybrid assembly mode of Unicycler version 0.4.1 [52]. Each genome was assembled into a single chromosome and plasmid contig. The genome sequences were annotated using the Rapid Annotations via Prokka—Prokaryotic genome annotation (Galaxy Version 1.14.6) [53,54,55]. Default parameters were used for all software unless otherwise specified.

### 4.5. Genome Analysis

#### 4.5.1. 16S rRNA and 23S rRNA Phylogenetic Analyses

The 16S and 23S rRNA gene sequences were extracted from avian *C. abortus* 15-70d24, 15-49d3, 15-58d44 genomes and representatives of family *Chlamydiaceae*, then aligned using MAFFTv7.013 by Geneious Pro 8.0 software (Biomatters, Auckland, New Zealand). Dendrograms were constructed by IQ-TREE v1.6.12 [56], with the best-fit model according to Bayesian Information Criterion (BIC) model and 1000 bootstrap replicates. The models were calculated by Model Finder of IQ-TREE v1.6.12 [57,58,59] and MEGA X [60,61]. The phylogeny was visualized by ITOL [62].

#### 4.5.2. Molecular Typing of Avian *C. abortus* Strains

The genetic relationship of avian *C. abortus* strains described in this study and other chlamydial species was assessed using the classification system published by Pillonel et al. [24] and recommended by the International Committee on Systematics of Prokaryotes Subcommittee on the taxonomy of Chlamydiae [4,46]. Nine phylogenetically informative markers were identified and extracted from 15 reference strains of *Chlamydia* spp., three *Candidatus Chlamydia* spp. (available in NCBI database) and one *Simaniaceae* representative. Amino acid sequences were aligned with MAFFTv7.013 by Geneious Pro 8.0 software (Biomatters, Auckland, New Zealand). Alignments were concatenated to build a reference phylogeny using IQ-TREE v1.6.12 [56], with the best-fit model according to BIC and 1000 bootstrap replicates. The model was calculated by Model Finder of IQ-TREE v1.6.12 [57,58,59] and MEGA X [60,61]. The phylogeny was visualized by ITOL [62]. Pairwise amino acid sequence identities were determined based on the MAFFT alignment by Geneious Pro 8.0 software (Biomatters, Auckland, New Zealand).

#### 4.5.3. ANIb and Tetra-Nucleotide Signatures

The Average Nucleotide Identity (ANIb) and correlation indexes of their Tetra-nucleotide signatures were calculated based on the program JSpecies v3.7.2 with default parameters for avian *C. abortus* strains 15-70d24, 15-49d3, 15-58d44 and selected chlamydial representatives [63].

#### 4.5.4. Plasmid Comparisons

Phylogenetic analysis based on plasmid sequences was performed on the 15-70d24, 15-49d3 and 15-58d44 strains and other *Chlamydiaceae* members (available in NCBI database), including *C. abortus* 84/2334 and *C. psittaci* genotypes A, B, D, E, E/B, F, M56 and WC. Nucleotide sequences were aligned using MAFFT in Geneious Pro 8.0 software (Biomatters, Auckland, New Zealand) and a phylogenetic tree was constructed using IQ-TREE v1.6.12 [56], with the best-fit model according to BIC and 1000 bootstrap replicates. The model was calculated by Model Finder of IQ-TREE v1.6.12 [57,58,59] and MEGA X [60,61]. The generated tree was visualized using ITOL [62]. Chlamydial plasmids arrangement was constructed using EasyFig [64].

#### 4.5.5. Plasticity Zone

In order to further characterise the genomes of avian *Chlamydia abortus*, the plasticity zone regions were analysed in comparison to other chlamydial species. PZs were extracted from *Chlamydia* spp. reference strains and avian *C. abortus* strains 15-70d24, 15-49d3, 15-58d44 (G1, G2, 1V, respectively) by Geneious Pro 8.0 software (Biomatters, Auckland, New Zealand). The visualisation of the BLAST comparisons were generated using Easyfig [64].

#### 4.5.6. Phylogenetic Network Analysis

Whole-genome sequences of *C. abortus*, *C. psittaci* (genotypes A, B, C, D, E, E/B, F, M56 and WC), *C. buteonis* and avian *C. abortus* G1, G2, 1V representative strains were aligned using MAFFT. The generated alignment in fasta format was imported into SplitsTree (v4.16.2) to generate a NeighborNet tree [65].

#### 4.5.7. SNP Analysis

Analysis included representatives of *C. abortus*, *C. psittaci* (genotypes A, B, C, D, E, E/B, F, M56 and WC), *C. buteonis* and avian *C. abortus* strains 15-70d24, 15-49d3, 15-58d44 (G1, G2, 1V, respectively). A representative batch of sequenced reads for each genome were taken from the Sequence Read Archive. All available next generation sequencing reads were quality checked and aligned against the reference genome (*C. abortus* 84/2334) using Burrows-Wheeler Aligner (BWA-MEM) (v. 0.7.17) [66]. Variant calling was performed using FreeBayes (v. 1.3.2) [67], while called variants were filtered by VcfFilter, part of the VCFlib (v. 1.0.2) [68]. SNP detection on downloaded assemblies were conducted using NUCmer (v.3.1) and Show-SNPs, in the MUMmer package [69]. Bcftools (v. 1.9) [70] was applied to combined the vcf files containing SNPs for each strain together. VCF-kit (v.0.2.9) was utilized to generate a single file in fasta format to be used for the construction of a phylogenetic tree using IQ-TREE v1.6.12 [56], with the best-fit model according to BIC and 1000 bootstrap replicates. The model was calculated by Model Finder of IQ-TREE v1.6.12 [57,58,59] and MEGA X [60,61]. The generated tree was visualized using ITOL [62]. As *C. abortus* 84/2334 is closely related to the sequenced samples it was used as a reference genome.

## Data Availability

The genome sequences of strains: 15-70d24, 15-49d3 and 15-58d44 obtained in this study have been deposited at ENA/GenBank/DDBJ under the accession numbers: LS450958.2, LS450956.2, OU508367.1 (chromosomes); LS450959.2, LS450957.2, OU508368.1 (plasmids), respectively. The Nanopore raw reads have been deposited under accession numbers: ERR6415086, ERR6415087, ERR6415088, respectively. All data related to WGS of avian *C. abortus* strains 15-70d24, 15-49d3 and 15-58d44 are included as a part of BioProject PRJEB26715.

## Supplementary Materials

The following are available online at https://www.mdpi.com/article/10.3390/pathogens10111405/s1, Table S1. Basic genomic parameters of avian *C. abortus* strains and selected representatives of *Chlamydia* spp. Table S2. Lower triangle represents pairwise sequence identity values for 16S rRNA, while upper triangle shows pairwise sequence identity values for 23S rRNA genes extracted from selected representatives of *Chlamydia* spp., *Candidatus* and avian *C. abortus* strains. Tables S3–5. Nine taxonomically conserved protein sequences extracted from the avian *C. abortus* strains 15-70d24, 15-49d3 and 15-58d44 (G1, G2 and 1V, respectively) compared to strains belonging to *Chlamydia* spp., *Candidatus* to *Chlamydia* species and *Simkania negevensis* Z. Red cells present identity values higher than the defined threshold values, whereas green cells show identity values below the threshold values. Table S6. Pairwise comparison of selected representatives of *Chlamydia* spp. and avian *C. abortus* strains. Lower triangle represents pairwise sequence identity values for tetranucleotide signature correlation index, while upper triangle shows pairwise sequence identity values for average nucleotide identity (ANIb). Table S7. Nucleotide identity values (%) of plasmid sequences of selected *Chlamydia* spp. Distance matrices based on multiple sequence alignment were used for calculation of nucleotide sequence identity values (Geneious Pro 8.0 software; Biomatters, Auckland, New Zealand). Table S8. Nucleotide identity values (%) of plasticity zone sequences of selected representative strains of *Chlamydia* spp. Distance matrices based on multiple sequence alignment were used for calculation of nucleotide sequence identity values (Geneious Pro 8.0 software; Biomatters, Auckland, New Zealand). Table S9. The number of SNP differences of selected representatives of *Chlamydia* spp. Table S10. Characterization of avian *C. abortus* strains. Table S11. Strains used in this study and their GenBank accession numbers.
