# Supplementary material for: Whole Genome Sequencing and Comparative Genome Analyses of Chlamydia abortus Strains of Avian Origin Suggests That Chlamydia abortus Species Should Be Expanded to Include Avian and Mammalian Subgroups
Source: Pathogens. 2021 Oct 29;10(11):1405. doi: 10.3390/pathogens10111405 (PMC8623937; doi:10.3390/pathogens10111405)
Supplement: Supplementary file 1 [file pathogens-10-01405-s001.zip › pathogens-1399461-supplementary.pdf]

	**Feature**	**avian *C. abortus***			***C. abortus* 84/2334**	***C. abortus* S26/3**	***C. psittaci* 6BC**	***C. poikilothermis* S15-834K**	***C. caviae* GPIC**	***C. felis* Fe/C-56**	***C. avium* 10DC88**	***C. gallinacea* 08-1274/3**	***C. pneumoniae* LPCoLN**	***C. serpentis* H15-1957-10C**	***C. pecorum* E58**	***C. muridarum* Nigg**	***C. trachomatis* A/HAR-13**	***C. suis* SWA-14**
		**15-70d24**	**15-49d3**	**15-58d44**														
		**genotype G1**	**genotype G2**	**genotype 1V**														
**Chromosome**	**Host**	Eurasian teal	mallard	magpie	parrot	sheep	parakeet	snake	guinea pig	cat	psittacine	chicken	koala	snake	cattle	mouse	human	pig
	**Length (bp)**	1,141,680	1,132,330	1,151,406	1,165,692	1,144,377	1,171,660	1,155,104	1,173,390	1,166,239	1,041,170	1,059,583	1,241,020	1,198,609	1,106,197	1,072,950	1,044,459	1,086,692
	**G+C content (%)**	39.9	39.6	39.8	39.9	39.9	39.1	37.5	39.2	39.4	36.9	37.9	40.5	37.5	41.1	40.3	41.3	42.1
	**No. of CDS**	992	992	1002	1005	1002	984	995	989	981	945	904	1028	1001	938	887	903	897
	**rRNA genes**	3	3	3	3	3	3	3	3	3	2	3	3	3	3	6	6	6
	**tRNA**	38	38	38	38	38	38	38	38	38	39	39	38	38	38	37	37	37
	**tmRNA**	1	1	1	1	1	1	1	1	1	1	1	1	1	1	1	1	1
**Plasmid**	**Length (bp)**	7553	7556	7553	7553	-	7553	7559	7966	7552	7099	7619	7530	7533	-	7501	7510	7494
	**G+C content (%)**	33.0	32.6	33.0	33.1	-	32.9	33.0	33.7	33.9	31.2	31.7	33.0	31.1	-	35.7	36.3	36.9
	**No. of ORFs**	8	8	8	8	-	8	8	8	8	8	9	8	8	-	8	8	8

Table S1. Basic genomic parameters of avian *C. abortus* strains and selected representatives of *Chlamydia* spp.

	avian *C. abortus* 15-70d24 (G1)	avian *C. abortus* 15-49d3 (G2)	avian *C. abortus* 15-58d44 (1V)	*C. abortus* 84/2334	*C. abortus* S26/3	*C. psittaci* 6BC	*C. buteonis* RSHA	*C. caviae* GPIC	*C. poikilothermis* S15-834K	*C. felis* Fe/C-56	*C. avium* 10DC88	*C. gallinacea* 08-1274/3	*Cand.* C. ibidis 10-1398/6	*C. pecorum* E58	*C. pneumoniae* LPCoLN	*C. serpentis* H15-1957-10C	*Cand.* C. corallus G3/2742-324	*Cand.* C. sanzinia G4/2742-308	*C. muridarum* Nigg	*C. trachomatis* A/HAR-13	*C. suis* SWA-14
**avian *C. abortus* 15-70d24 (G1)**	-	99.42	99.66	99.80	99.59	99.49	99.49	98.09	98.19	98.23	96.63	96.25	96.46	96.05	96.97	97.17	96.80	96.01	93.09	92.24	92.55
**avian *C. abortus* 15-49d3 (G2)**	99.35	-	99.56	99.63	99.42	99.45	99.35	98.13	98.19	98.19	96.63	96.25	96.29	95.84	96.93	97.07	96.80	96.05	93.12	92.17	92.62
**avian *C. abortus* 15-58d44 (1V)**	99.61	99.10	-	99.86	99.73	99.63	99.59	98.23	98.30	98.36	96.70	96.39	96.46	96.12	97.10	97.24	96.90	96.29	93.12	92.24	92.55
***C. abortus* 84/2334**	99.94	99.42	99.68	-	99.80	99.69	99.66	98.30	98.36	98.43	96.76	96.39	96.52	96.15	97.17	97.31	96.97	96.22	93.19	92.31	92.62
***C. abortus* S26/3**	99.81	99.29	99.55	99.87	-	99.49	99.45	98.09	98.16	98.23	96.63	96.39	96.32	95.98	96.97	97.10	96.76	96.08	93.12	92.24	92.55
***C. psittaci* 6BC**	99.74	99.48	99.48	99.81	99.68	-	99.69	98.40	98.53	98.53	96.63	96.46	96.59	96.08	97.17	97.31	97.04	96.35	93.33	92.41	92.72
***C. buteonis* RSHA**	99.61	99.10	99.35	99.68	99.55	99.61	-	98.30	98.36	98.53	96.73	96.32	96.63	96.29	97.34	97.48	97.14	96.32	93.26	92.31	92.62
***C. caviae* GPIC**	99.35	99.10	99.10	99.42	99.29	99.22	99.35	-	99.11	98.50	96.05	95.94	96.15	95.40	96.73	96.87	96.80	95.88	93.02	92.34	92.65
***C. poikilothermis* S15-834K**	99.35	99.03	99.10	99.42	99.29	99.35	99.35	99.48	-	98.67	96.08	95.95	96.29	95.44	96.90	97.10	97.04	96.22	92.85	92.21	92.38
***C. felis* Fe/C-58**	98.39	98.26	98.13	98.45	98.32	98.64	98.51	98.51	98.39	-	96.22	95.98	96.42	95.57	96.87	97.00	96.90	96.15	92.79	92.11	92.38
***C. avium* 10DC88**	97.67	97.48	97.48	97.74	97.61	97.80	97.67	97.74	97.67	96.96	-	98.71	95.37	95.43	96.12	96.22	96.12	95.13	92.99	92.14	92.31
***C. gallinacea* 08-1274/3**	97.80	97.61	97.67	97.87	97.74	97.93	97.55	97.48	97.42	96.90	98.38	-	95.23	95.09	95.91	95.95	95.95	95.37	92.82	92.07	92.34
***Cand.* C. ibidis 10-1398/6**	96.97	96.71	96.71	97.03	96.90	97.09	96.97	96.90	96.77	96.51	95.80	96.45	-	95.64	96.08	96.39	96.25	95.74	93.13	92.65	92.78
***C. pecorum* E58**	96.19	96.06	96.06	96.26	96.13	96.32	96.19	96.13	96.13	95.87	95.81	95.81	96.58	-	96.28	96.39	96.39	95.98	92.48	91.69	92.07
***C. pneumoniae* LPCoLN**	96.06	95.81	95.94	96.13	96.00	96.32	95.94	95.74	95.87	95.48	94.71	94.71	96.52	96.00	-	99.35	99.11	97.65	93.70	92.81	93.12
***C. serpentis* H15-1957-10C**	96.26	96.00	96.13	96.32	96.19	96.52	96.13	95.94	96.06	95.68	94.90	94.90	96.71	96.19	99.55	-	99.15	97.75	93.77	92.85	93.09
***Cand.* C. corallus G3/2742-324**	96.13	95.87	96.00	96.19	96.06	96.39	96.00	95.81	95.94	95.42	94.77	94.77	96.39	95.87	99.23	99.68	-	97.68	93.63	92.75	93.05
***Cand.* C. sanzinia G4/2742-308**	95.61	95.48	95.35	95.67	95.55	95.87	95.61	95.93	95.80	95.42	95.03	94.51	96.39	96.39	97.23	97.29	97.10	-	93.09	92.17	92.41
***C. muridarum* Nigg**	95.80	95.55	95.55	95.74	95.61	95.67	95.61	95.87	95.74	95.67	95.03	94.58	95.80	95.29	94.65	94.71	94.39	94.65	-	97.65	97.79
***C. trachomatis* A/HAR-13**	95.10	95.03	94.84	95.03	94.90	94.97	94.90	95.16	95.16	94.84	94.58	94.13	95.03	94.72	93.81	94.01	93.75	93.94	98.26	-	98.16
***C. suis* SWA-14**	94.19	94.13	93.93	94.13	94.00	94.19	94.00	94.32	94.13	94.06	94.18	93.73	93.80	93.49	92.59	92.78	92.78	93.16	97.03	96.65	-

Table S2. Lower triangle represents pairwise sequence identity values for 16S rRNA, while upper triangle shows pairwise sequence identity values for 23S rRNA genes extracted from selected representatives of *Chlamydia* spp., *Candidatus* and avian *C. abortus* strains.

Threshold		avian *C. abortus* 15-70d24 (G1)		Whole genome reference strains																				
		Gene	Length	avian *C. abortus* 15-58d44 (1V)	avian *C. abortus* 15-49d3 (G2)	*C. abortus* 84/2334	*C. abortus* S26/3	*C. psittaci* 6BC	*C. buteonis* RSHA	*C. poikilothermis* S15-834K	*C. caviae* GPIC	*C. felis* Fe/C-56	*C. avium* 10DC88	*C. gallinacea* 08-1274/3	*Cand.* C. ibidis 10-1398/6	*C. pneumoniae* LPCoLN	*Cand.* C. corallus G3/2742-324	*C. serpentis* H15-1957-10C	*Cand.* C. sanzinia G4/2742-308	*C. pecorum* E58	*C. muridarum* Nigg	*C. trachomatis* A/HAR-13	*C. suis* SWA-14	*Simkania negevensis* Z
Genus	70%	DnaA	460 aa	99.13	99.13	99.35	99.13	98.26	98.70	92.83	93.48	94.35	85.22	81.74	82.61	81.30	80.39	80.43	78.26	78.04	78.99	79.43	79.21	36.32
	78%	FabI	299 aa	99.67	99.33	99.67	99.67	93.31	96.99	92.31	91.97	90.64	89.30	87.29	82.61	84.62	85.28	85.28	82.94	84.90	84.56	83.22	83.89	66.56
	57%	Hyp325	425 aa	99.53	99.29	99.76	99.53	98.35	98.12	92.94	90.59	90.59	78.82	78.55	75.35	73.13	72.66	70.02	72.37	67.84	65.18	64.71	63.06	34.19
	64%	SucA	919 aa	99.12	98.79	99.56	99.34	96.04	96.37	91.30	90.20	89.10	78.33	77.89	76.13	73.27	73.60	73.49	74.75	69.54	69.16	68.17	67.95	39.45
Species	96%	RpoN	423 aa	97.87	97.40	98.11	97.87	94.33	92.91	84.63	84.16	83.45	66.04	65.48	59.20	58.45	58.22	57.01	59.72	57.65	47.76	46.82	49.65	26.81
	96%	PepF	608 aa	98.52	98.19	98.85	98.85	94.57	95.72	86.86	87.19	81.91	77.80	75.66	69.24	66.78	68.09	66.28	66.61	66.94	64.43	64.10	65.25	39.90
	95%	Adk	213 aa	99.06	95.31	98.59	98.59	92.02	91.55	75.59	75.23	71.36	62.20	63.64	49.77	52.58	52.11	53.05	49.77	53.52	44.69	45.13	43.36	34.56
	98%	FtsK	804 aa	98.76	98.38	99.27	98.88	96.39	95.27	90.31	90.06	90.68	80.00	79.53	78.29	70.76	71.13	70.76	71.06	69.63	70.73	71.59	69.79	44.70
	95%	HemL	437 aa	99.31	99.31	99.31	99.54	91.76	92.68	78.03	77.35	78.72	65.44	64.52	58.85	55.61	55.61	55.38	58.24	54.46	55.09	53.24	53.47	36.93

Table S3-S5. Nine taxonomically conserved protein sequences extracted from the avian *C. abortus* strains 15-70d24, 15-49d3 and 15-58d44 (G1, G2 and 1V, respectively) compared to strains belonging to *Chlamydia* spp., *Candidatus* to *Chlamydia* species and *Simkania negevensis* Z. Red cells present identity values higher than the defined threshold values, whereas green cells show identity values below the threshold values.

Threshold		avian *C. abortus* 15-49d3 (G2)		Whole genome reference strains																				
		Gene	Length	avian *C. abortus* 15-70d24 (G1)	avian *C. abortus* 15-58d44 (1V)	*C. abortus* 84/2334	*C. abortus* S26/3	*C. psittaci* 6BC	*C. buteonis* RSHA	*C. poikilothermis* S15-834K	*C. caviae* GPIC	*C. felis* Fe/C-56	*C. avium* 10DC88	*C. gallinacea* 08-1274/3	*Cand.* C. ibidis 10-1398/6	*C. pneumoniae* LPCoLN	*Cand.* C. corallus G3/2742-324	*C. serpentis* H15-1957-10C	*Cand.* C. sanzinia G4/2742-308	*C. pecorum* E58	*C. muridarum* Nigg	*C. trachomatis* A/HAR-13	*C. suis* SWA-14	*Simkania negevensis* Z
**Genus**	**70%**	**DnaA**	460 aa	99.13	99.57	99.78	99.57	98.70	99.13	93.26	93.91	94.78	85.22	81.74	82.83	81.52	80.61	80.65	78.26	78.26	79.21	79.43	79.21	36.32
	**78%**	**FabI**	299 aa	99.33	99.67	99.67	99.67	93.31	96.99	92.31	91.97	90.64	89.30	87.29	82.61	84.62	85.28	85.28	82.94	84.90	85.23	82.55	83.89	66.56
	**57%**	**Hyp325**	425 aa	99.29	98.82	99.06	98.82	97.65	97.41	92.24	90.12	89.88	78.12	77.86	74.88	73.36	72.90	70.02	72.37	67.84	65.18	64.24	63.06	34.19
	**64%**	**SucA**	908 aa	98.79	98.79	99.01	98.79	95.59	95.93	90.86	89.87	88.66	77.78	77.78	76.02	73.38	73.49	73.60	74.86	69.76	69.49	68.50	68.17	39.67
**Species**	**96%**	**RpoN**	423 aa	97.40	97.64	98.11	97.64	95.51	94.09	85.34	84.87	83.92	66.51	67.14	59.67	59.15	58.45	57.94	60.42	57.65	48.71	47.29	50.12	26.34
	**96%**	**PepF**	608 aa	98.19	98.03	98.36	98.36	93.42	94.57	86.04	86.37	81.09	77.14	75.16	68.59	66.28	67.60	65.63	66.12	66.12	64.26	64.10	65.25	39.74
	**95%**	**Adk**	220 aa	95.31	95.77	95.77	95.31	89.20	89.67	73.15	71.95	69.95	61.72	62.20	49.53	51.64	51.64	51.64	48.18	54.46	44.93	44.93	44.05	33.48
	**98%**	**FtsK**	804 aa	98.38	98.38	98.91	98.51	96.39	95.40	90.56	90.31	90.68	79.88	79.90	78.16	70.63	70.63	70.26	70.81	69.75	70.73	71.46	69.91	44.83
	**95%**	**HemL**	437 aa	99.31	99.08	99.08	99.31	91.53	92.45	78.26	77.57	78.49	65.21	64.52	58.85	55.38	55.38	55.15	58.24	54.23	55.09	53.01	53.47	36.93

Table S3-S5. Nine taxonomically conserved protein sequences extracted from the avian *C. abortus* strains 15-70d24, 15-49d3 and 15-58d44 (G1, G2 and 1V, respectively) compared to strains belonging to *Chlamydia* spp., *Candidatus* to *Chlamydia* species and *Simkania negevensis* Z. Red cells present identity values higher than the defined threshold values, whereas green cells show identity values below the threshold values.

Threshold		avian *C. abortus* 15-58d44 (1V)		Whole genome reference strains																				
		Gene	Length	avian *C. abortus* 15-70d24 (G1)	avian *C. abortus* 15-49d3 (G2)	*C. abortus* 84/2334	*C. abortus* S26/3	*C. psittaci* 6BC	*C. buteonis* RSHA	*C. poikilothermis* S15-834K	*C. caviae* GPIC	*C. felis* Fe/C-56	*C. avium* 10DC88	*C. gallinacea* 08-1274/3	*Cand.* C. ibidis 10-1398/6	*C. pneumoniae* LPCoLN	*Cand.* C. corallus G3/2742-324	*C. serpentis* H15-1957-10C	*Cand.* C. sanzinia G4/2742-308	*C. pecorum* E58	*C. muridarum* Nigg	*C. trachomatis* A/HAR-13	*C. suis* SWA-14	*Simkania negevensis* Z
Genus	70%	DnaA	460 aa	99.13	99.57	99.78	99.57	98.70	99.13	93.26	93.91	95.00	85.22	81.74	82.83	81.74	80.61	80.87	78.48	78.48	79.21	79.43	79.21	36.32
	78%	Fabl	299 aa	99.67	99.67	100	100	93.65	97.32	92.64	92.31	90.97	89.63	87.63	82.94	84.95	85.62	85.62	83.28	85.23	84.90	82.89	84.23	66.89
	57%	Hyp325	425 aa	99.53	98.82	99.29	99.06	97.88	97.65	92.47	90.12	90.12	78.59	78.55	75.35	72.90	72.66	70.02	72.37	67.61	64.94	64.94	63.29	34.19
	64%	SucA	908 aa	99.12	98.79	99.34	99.12	96.04	96.26	91.30	90.20	89.32	78.22	77.89	76.02	73.27	73.71	73.49	74.86	69.76	69.38	68.39	68.17	39.67
Species	96%	RpoN	423 aa	97.87	97.64	98.82	98.58	94.09	93.14	85.11	84.63	83.92	66.04	66.19	59.43	58.45	58.22	57.01	60.42	57.65	48.24	47.06	50.12	26.81
	96%	PepF	608 aa	98.52	98.03	99.34	99.34	93.75	94.90	86.21	86.54	81.25	76.97	74.84	68.75	66.28	67.93	65.79	66.28	66.28	64.75	64.26	65.90	39.57
	95%	Adk	213 aa	99.06	95.77	99.53	99.53	92.02	92.49	76.53	75.70	71.83	62.68	64.11	50.23	52.58	52.11	53.05	49.30	53.99	45.13	45.58	43.81	35.48
	98%	FtsK	804 aa	98.76	98.38	99.27	98.63	96.52	95.52	90.31	90.06	90.68	79.75	79.28	77.92	70.51	70.63	70.38	70.93	69.63	70.85	71.46	69.91	44.83
	95%	HemL	437 aa	99.31	99.08	99.08	99.31	91.53	92.91	78.03	77.12	78.26	64.98	64.06	58.62	55.38	55.38	55.15	58.47	54.23	55.32	53.47	53.47	37.16

Table S3-S5. Nine taxonomically conserved protein sequences extracted from the avian *C. abortus* strains 15-70d24, 15-49d3 and 15-58d44 (G1, G2 and 1V, respectively) compared to strains belonging to *Chlamydia* spp., *Candidatus* to *Chlamydia* species and *Simkania negevensis* Z. Red cells present identity values higher than the defined threshold values, whereas green cells show identity values below the threshold values.

Legend:

values above the threshold

values below the threshold

	avian *C. abortus* 15-70d24 (G1)	avian *C. abortus* 15-49d3 (G2)	avian *C. abortus* 15-58d44 (1V)	*C. abortus* 84/2334	*C. abortus* S26/3	*C. psittaci* 6BC	*C. buteonis* RSHA	*C. caviae* GPIC	*C. felis* Fe/C-56	*C. avium* 10DC88	*C. gallinacea* 08-1274/3	*C. pneumoniae* LPCoLN	*C. pecorum* E58	*C. muridarum* Nigg	*C. trachomatis* A/HAR-13
**avian *C. abortus* 15-70d24 (G1)**	-	97.84	97.81	98.07	97.93	92.20	92.42	80.66	80.32	73.57	73.21	69.70	68.98	68.48	68.47
**avian *C. abortus* 15-49d3 (G2)**	0.99845	-	97.23	97.47	97.47	92.14	92.34	80.56	80.25	73.72	73.28	69.75	68.95	68.31	68.28
**avian *C. abortus* 15-58d44 (1V)**	0.99878	0.99808	-	98.73	98.36	92.46	92.27	80.52	80.24	73.71	73.30	69.83	69.11	68.55	68.51
***C. abortus* 84/2334**	0.99851	0.99795	0.99903	-	98.92	92.68	92.37	80.64	80.22	73.69	73.29	69.77	68.99	68.52	68.39
***C. abortus* S26/3**	0.99881	0.99783	0.9989	0.99913	-	92.54	92.48	80.64	80.21	73.63	73.46	69.84	69.14	68.65	68.59
***C. psittaci* 6BC**	0.98791	0.98905	0.98773	0.98646	0.98585	-	93.40	80.98	80.59	73.93	73.42	69.92	69.11	68.71	68.52
***C. buteonis* RSHA**	0.96938	0.97309	0.96843	0.96628	0.96631	0.9874	-	81.41	81.07	74.02	73.61	69.94	69.10	68.49	68.45
***C. caviae* GPIC**	0.95876	0.95797	0.95983	0.95771	0.95704	0.97015	0.96605	-	81.81	74.10	73.76	70.06	69.41	68.81	68.60
***C. felis* Fe/C-56**	0.96041	0.96469	0.95879	0.95617	0.95665	0.9663	0.97045	0.95143	-	73.78	73.47	69.96	68.92	68.46	68.30
***C. avium* 10DC88**	0.90137	0.91304	0.89959	0.89893	0.89832	0.92367	0.93948	0.89991	0.92731	-	81.15	69.44	68.98	68.68	68.51
***C. gallinacea* 08-1274/3**	0.92814	0.9374	0.92524	0.92356	0.92393	0.94173	0.94845	0.91187	0.94793	0.98642	-	69.28	68.73	68.43	68.32
***C. pneumoniae* LPCoLN**	0.90143	0.89826	0.90233	0.90184	0.90352	0.89521	0.86954	0.91119	0.87639	0.80706	0.83060	-	69.61	67.58	67.65
***C. pecorum* E58**	0.88269	0.88284	0.88567	088429	0.88324	0.88236	0.85674	0.89201	0.87343	0.81059	0.83297	0.90213	-	67.43	67.20
***C. muridarum* Nigg**	0.83240	0.84097	0.83835	0.83694	0.83601	0.85406	0.8549	0.84306	0.8423	0.80527	0.82163	0.79774	0.77658	-	80.21
***C. trachomatis* A/HAR-13**	0.83202	0.83927	0.83984	0.83723	0.83561	0.84321	0.8319	0.83390	0.8299	0.76501	0.78840	0.79042	0.79041	0.97216	-

Table S6. Pairwise comparison of selected representatives of *Chlamydia* spp. and avian *C. abortus* strains. Lower triangle represents pairwise sequence identity values for tetranucleotide signature correlation index, while upper triangle shows pairwise sequence identity values for average nucleotide identity (ANIb).

Legend:
Upper tringle - Above cutoff (> 95%) Below cutoff (< 95%)
Lower tringle - Above cutoff (> 0.999) In range (> 0.989) Below cutoff (< 0.989)

	avian *C. abortus* 15-70d24 (G1)	avian *C. abortus* 15-49d3 (G2)	avian *C. abortus* 15-58d44 (1V)	*C. abortus* 84/2334	*C. psittaci* 6BC (genotype A)	*C. psittaci* CP3 (genotype B)	*C. psittaci* NJ1 (genotype D)	*C. psittaci* MN (genotype E)	*C. psittaci* WS/RT/E30 (genotype E/B)	*C. psittaci* VS2255 (genotype F)	*C. psittaci* M56 (genotype M56)	*C. psittaci* WC (genotype WC)	*C. caviae* GPIC	*C. poikilothermis* S15-834K	*C. felis* Fe/C-56	*C. gallinacea* 08-1274/3	*C. avium* 10DC88	*C. pneumoniae* LPCoLN	*Cand.* C. sanzinia G4/2742-308	*C. serpentis* H15-1957-10C	*Cand.* C. corallus G3/2742-324	*C. pecorum* L1	*C. muridarum* Nigg	*C. suis* SWA-14	*C. trachomatis* A/HAR-13
**avian *C. abortus* 15-70d24 (G1)**	-	98.73	97.43	97.63	94.61	95.71	95.71	94.86	95.62	95.62	95.51	95.68	81.81	82.28	79.85	72.88	70.74	68.41	68.28	67.92	68.21	67.16	59.33	59.13	58.69
**avian *C. abortus* 15-49d3 (G2)**	98.73	-	97.13	97.33	94.48	95.46	95.39	94.73	95.47	95.47	95.22	95.43	81.59	82.18	79.69	72.80	70.64	68.60	68.48	67.87	68.17	67.17	59.41	59.20	58.82
**avian *C. abortus* 15-58d44 (1V)**	97.43	97.13	-	99.54	94.68	95.75	95.70	94.89	95.66	95.66	95.58	95.72	81.77	82.40	79.81	72.81	70.55	68.31	68.34	67.71	68.23	67.06	58.95	59.16	58.66

Table S7. Nucleotide identity values (%) of plasmid sequences of selected *Chlamydia* spp. Distance matrices based on multiple sequence alignment were used for calculation of nucleotide sequence identity values (Geneious Pro 8.0 software; Biomatters, Auckland, New Zealand).

	avian *C. abortus* 15-70d24 (G1)	avian *C. abortus* 15-49d3 (G2)	avian *C. abortus* 15-58d44 (1V)	***C. abortus*** **84/2334**	***C. buteonis*** **RSHA**	***C. psittaci*** **6BC**	***Cand.*** **C. ibidis 10-1398/6**	***C. abortus*** **S26/3**	***C. gallinacea*** **08-1274/3**	***C. caviae*** **GPIC**	***C. felis*** **Fe/C-56**	***Cand.*** **C. corallus G3/2742-324**	***C. pneumoniae*** **AR39**	***C. avium*** **10DC88**	***C. poikilothermis*** **S15-834K**	***Cand.*** **C. sanzinia G4/2742-308**	***C. trachomatis*** **D/UW-3/CX**	***C. muridarum*** **Nigg**	***C. suis*** **SWA-14**
**avian *C. abortus* 15-70d24 (G1)**	-	91.21	56.70	51.5	62.06	49.12	41.93	25.54	25.51	25.67	21.35	20.79	19.53	16.39	13.77	13.46	13.68	9.38	9.10
**avian *C. abortus* 15-49d3 (G2)**	91.21	-	69.47	69.09	70.85	64.19	55.60	32.98	28.46	33.32	24.81	22.90	21.42	16.23	17.47	15.20	17.68	17.67	16.95
**avian *C. abortus* 15-58d44 (1V)**	56.70	69.47	-	77.67	54.47	62.65	39.89	38.35	26.38	31.24	22.98	21.25	19.78	15.65	17.24	14.73	14.72	10.53	9.79

Table S8. Nucleotide identity values (%) of plasticity zone sequences of selected representative strains of *Chlamydia* spp. Distance matrices based on multiple sequence alignment were used for calculation of nucleotide sequence identity values (Geneious Pro 8.0 software; Biomatters, Auckland, New Zealand).

	avian *C. abortus* 15-70d24 (G1)	avian *C. abortus* 15-49d3 (G2)	avian *C. abortus* 15-58d44 (1V)	*C. abortus* 84/2334	*C. abortus* S26/3	*C. abortus* AB7	*C. abortus* B577	*C. abortus* LLG	*C. psittaci* 6BC (genotype A)	*C. psittaci* CP3 (genotype B)	*C. psittaci* GR9 (genotype C)	*C. psittaci* NJ1 (genotype D)	*C. psittaci* MN (genotype E)	*C. psittaci* WS/RT/E30 (genotype E/B)	*C. psittaci* VS2255 (genotype F)	*C. psittaci* M56 (genotype M56)	*C. psittaci* WC (genotype WC)	*C. buteonis* RSHA
**avian *C. abortus* 15-70d24 (G1)**	-	15,562	13,071	12,126	11,957	12,084	12,020	12,637	25,107	25,006	23,960	25,032	28,112	68,907	25,190	28,405	31,264	72,856
**avian *C. abortus* 15-49d3 (G2)**	15,562	-	16,891	16,328	16,064	16,185	16,142	16,730	28,590	28,493	27,105	28,535	31,629	69,925	28,680	31,936	34,814	74,315
**avian *C. abortus* 15-58d44 (1V)**	13,071	16,891	-	8892	8872	8996	8957	9554	22,573	22,460	21,854	22,530	25,579	68,768	22,675	25,897	28,739	72,206
***C. abortus* 84/2334**	12,126	16,328	8892	-	2074	2058	2032	2512	15,803	15,698	15,047	15,769	18,536	67,720	15,865	18,848	21,490	71,075
***C. abortus* S26/3**	11,957	16,064	8872	2074	-	251	120	1378	15,555	15,452	14,847	15,504	18,455	67,067	15,644	18,756	21,522	70,614
***C. abortus* AB7**	12,084	16,185	8996	2058	251	-	221	1392	15,530	15,431	14,822	15,480	18,407	67,201	15,615	18,717	21,460	70,748
***C. abortus* B577**	12,020	16,142	8957	2032	120	221	-	1365	15,497	15,399	14,802	15,427	18,375	67,171	15,566	18,691	21,434	70,720
***C. abortus* LLG**	12,637	16,730	9554	2512	1378	1392	1365	-	16,000	15,909	15,285	15,965	18,744	67,764	16,095	19,043	21,600	71,262
***C. psittaci* 6BC (genotype A)**	25,107	28,590	22,573	15,803	15,555	15,530	15,497	16,000	-	472	3151	2644	8572	56,327	1458	11,816	15,912	69,624
***C. psittaci* CP3 (genotype B)**	25,006	28,493	22,460	15,698	15,452	15,431	15,399	15,909	472	-	2951	2504	8667	56,072	1349	11,893	16,115	69,446
***C. psittaci* GR9 (genotype C)**	23,960	27,105	21,854	15,047	14,847	14,822	14,802	15,285	3151	2951	-	4243	9929	54,896	3381	12,486	16,596	70,044
***C. psittaci* NJ1 (genotype D)**	25,032	28,535	22,530	15,769	15,504	15,480	15,427	15,965	2644	2504	4243	-	9493	57,280	2868	12,241	16,649	69,625
***C. psittaci* MN (genotype E)**	28,112	31,629	25,579	18,536	18,455	18,407	18,375	18,744	8572	8667	9929	9493	-	63,412	8699	12,580	12,976	74,707
***C. psittaci* WS/RT/E30 (genotype E/B)**	68,907	69,925	68,768	67,720	67,067	67,201	67,171	67,764	56,327	56,072	54,896	57,280	63,412	-	56,649	66,189	70,408	60,903
***C. psittaci* VS2255 (genotype F)**	25,190	28,680	22,675	15,865	15,644	15,615	15,566	16,095	1458	1349	3381	2868	8699	56,649	-	11,798	15,801	69,828
***C. psittaci* M56 (genotype M56)**	28,405	31,936	25,897	18,848	18,756	18,717	18,691	19,043	11,816	11,893	12,486	12,241	12,580	66,189	11,796	-	13,372	74,142
***C. psittaci* WC (genotype WC)**	31,264	34,814	28,739	21,490	21,522	21,460	21,434	21,600	15,912	16,115	16,596	16,649	12,976	70,408	15,801	13,372	-	79,781
***C. buteonis* RSHA**	72,856	74,315	72,206	71,075	70,614	70,748	70,720	71,262	69,624	69,446	70,044	69,625	74,707	60,903	69,828	74,142	79,781	-

Table S9. The number of SNP differences of selected representatives of *Chlamydia* spp.

Designation	Host	Year	Genotype	MLST sequence type	GenBank accession number			
					**MLST (*gidA, enoA, fumC, gatA, hemN, hflx, oppA*)**	***omp*A**	**16S-23S ribosomal RNA intergenic spacer**	**WGS based on Illumina sequencing**
**15-70d24**	Eurasian teal *(Anas crecca)*	2015/ 2016	**G1**	ST154	KX870503<br>KX870475<br>KX870476<br>KX870477<br>KX870478<br>KX870479<br>KX870480<br>KX870481	KX870485	KX870503	chromosome:<br>LS450958.1<br>plasmid:<br>LS450959.1<br>raw reads:<br>ERS2484026.1
**15-49d3**	mallard *(Anas platyrhynchos)*	2015/ 2016	**G2**	ST151	KX870501<br>KX870454<br>KX870455<br>KX870456<br>KX870457<br>KX870458<br>KX870459<br>KX870460	KX870483	KX870501	chromosome:<br>LS4509561<br>plasmid:<br>LS450957.1<br>raw reads:<br>ERS2484025.1
**15-58d44**	magpie *(Pica pica)*	2015/ 2016	**1V**	ST152	KX870502<br>KX870461<br>KX870462<br>KX870463<br>KX870464<br>KX870465<br>KX870466<br>KX870467	KX870484	KX870502	chromosome:<br>CAJHTZ010000001.1<br>CAJHTZ010000002.1<br>CAJHTZ010000004.1<br>CAJHTZ010000005.1<br>plasmid:<br>CAJHTZ010000003.1<br>raw reads:<br>ERS2484027.1

Table S10. Characterization of avian *C. abortus* strains.

16S rRNA & 23S rRNA phylogenetic analyses	Molecular typing	ANIb & Tetra-nucleotide signatures	Pladsmid comparisons	Plasticity zone	Phylogenetic network analysis	SNP analysis
avian *C. abortus*<br>15-70d24<br>(LS450958.2)	avian *C. abortus*<br>15-70d24<br>(LS450958.2)	avian *C. abortus*<br>15-70d24<br>(LS450958.2; LS450959.2)	avian *C. abortus*<br>15-70d24<br>(LS450959.2)	avian *C. abortus*<br>15-70d24<br>(LS450958.2)	avian *C. abortus*<br>15-70d24<br>(LS450958.2; LS450959.2)	avian *C. abortus*<br>15-70d24<br>(ERR6415086.1)
avian *C. abortus*<br>15-49d3<br>(LS450956.2)	avian *C. abortus*<br>15-49d3<br>(LS450956.2)	avian *C. abortus*<br>15-49d3<br>(LS450956.2; LS450957.2)	avian *C. abortus*<br>15-49d3<br>(LS450957.2)	avian *C. abortus*<br>15-49d3<br>(LS450956.2)	avian *C. abortus*<br>15-49d3<br>(LS450956.2; LS450957.2)	avian *C. abortus*<br>15-49d3<br>(ERR6415087.1)
avian *C. abortus*<br>15-58d44<br>(OU508367.1)	avian *C. abortus*<br>15-58d44<br>(OU508367.1)	avian *C. abortus*<br>15-58d44<br>(OU508367.1; OU508368.1)	avian *C. abortus*<br>15-58d44<br>(OU508368.1)	avian *C. abortus*<br>15-58d44<br>(OU508367.1)	avian *C. abortus*<br>15-58d44<br>(OU508367.1; OU508368.1)	avian *C. abortus*<br>15-58d44<br>(ERR6415088.1)
C. abortus<br>84/2334<br>(CP031646.1)	*C. abortus*<br>84/2334<br>(CP031646.1)	*C. abortus*<br>84/2334<br>(CP031646.1; CP031647.1)	*C. abortus*<br>84/2334<br>(CP031647.1)	*C. abortus*<br>84/2334<br>(CP031646.1)	*C. abortus*<br>84/2334<br>(CP031646.1; CP031647.1)	*C. abortus*<br>84/2334<br>(SRR13156196.1)
C. abortus<br>S26/3<br>(CR848038.1)	*C. abortus*<br>S26/3<br>(CR848038.1)	*C. abortus*<br>S26/3<br>(GCA_000026025.1)	*C. avium*<br>10DC88<br>(CP006572.1)	*C. abortus*<br>S26/3<br>(CR848038.1)	*C. abortus*<br>AB7<br>(LN554882.1)	*C. abortus*<br>AB7<br>(ERR175610.1)
C. avium<br>10DC88<br>(CP006571.1)	*C. avium*<br>10DC88<br>(CP006571.1)	*C. avium*<br>10DC88<br>(GCA_000583875.1)	*C. caviae*<br>GPIC<br>(AE015926.1)	*C. avium*<br>10DC88<br>(CP006571.1)	*C. abortus*<br>B577<br>(CP024084.1)	*C. abortus*<br>B577<br>(SRR2089367.1)
C. buteonis<br>RSHA<br>(CAAAFM010000000.1)	*C. buteonis*<br>RSHA<br>(CAAAFM010000000.1)	*C. buteonis*<br>RSHA<br>(CAAAFM010000000.1)	*C. felis*<br>Fe/C-56<br>(AP006862.1)	*C. buteonis*<br>RSHA<br>(CAAAFM010000000.1)	*C. abortus*<br>LLG<br>(CP018296.1)	*C. abortus*<br>LLG<br>(ERR175609.1)
C. caviae<br>GPIC<br>(AE015925.1)	*C. caviae*<br>GPIC<br>(AE015925.1)	*C. caviae*<br>GPIC<br>(GCA_000007605.1)	*C. gallinacea*<br>08-1274/3<br>(CP015841.1)	*C. caviae*<br>GPIC<br>(AE015925.1)	*C. abortus*<br>S26/3<br>(CR848038.1)	*C. abortus*<br>S26/3<br>(NC_004552.2)
C. felis<br>Fe/C-56<br>(AP006861.1)	*C. felis*<br>Fe/C-56<br>(AP006861.1)	*C. felis*<br>Fe/C-56<br>(AP006861.1; AP006862.1)	*C. muridarum*<br>Nigg<br>(AE002162.1)	*C. felis*<br>Fe/C-56<br>(AP006861.1)	*C. buteonis*<br>RSHA<br>(CAAAFM010000000.1)	*C. buteonis*<br>RSHA<br>(GCF_900634605.1)
C. gallinacea<br>08-1274/3<br>(NZ_CP015840.1)	*C. gallinacea*<br>08-1274/3<br>(NZ_CP015840.1)	*C. gallinacea*<br>08-1274/3<br>(GCA_000471025.2)	*C. pecorum*<br>L1<br>(CM003639.1)	*C. gallinacea*<br>08-1274/3<br>(CP015840.1)	*C. psittaci*<br>6BC<br>(CP002586.1; CP002587.1)	*C. psittaci*<br>6BC<br>(SRR652458.1)
C. muridarum<br>Nigg<br>(AE002160.2)	*C. muridarum*<br>Nigg<br>(AE002160.2)	*C. muridarum*<br>Nigg<br>(GCA_000006685.1)	*C. pneumoniae*<br>LPCoLN<br>(CP001714.1)	*C. muridarum*<br>Nigg<br>(AE002160.1)	*C. psittaci*<br>CP3<br>(CP003797.1; CP003813.1)	*C. psittaci*<br>CP3<br>(SRR652460.1)
C. pecorum<br>E58<br>(CP002608.1)	*C. pecorum*<br>E58<br>(CP002608.1)	*C. pecorum*<br>E58<br>(GCA_000204135.1)	*C. poikilothermis*<br>S15-834C<br>(LS992155.1)	*C. pecorum*<br>E58<br>(CP002608.1)	*C. psittaci*<br>GR9<br>(CP003791.1)	*C. psittaci*<br>GR9<br>(SRR652456.1)
C. pneumoniae<br>LPCoLN<br>(CP001713.1)	*C. pneumoniae*<br>LPCoLN<br>(CP001713.1)	*C. psittaci*<br>6BC<br>(GCA_000191925.1)	*C. psittaci*<br>6BC<br>(CP002550.1)	*C. pneumoniae*<br>AR39<br>(AE002161.1)	*C. psittaci*<br>M56<br>(CP003795.1; CP003814.1)	*C. psittaci*<br>M56<br>(SRR652459.1)
C. poikilothermis<br>S15-834K<br>(LS992154.1)	*C. poikilothermis*<br>S15-834K<br>(LS992154.1)	*C. pneumoniae*<br>LPCoLN<br>(GCA_000024145.1)	*C. psittaci*<br>CP3<br>(CP003813.1)	*C. poikilothermis*<br>S15-834K<br>(LS992154.1)	*C. psittaci*<br>MN<br>(CP003792.1; CP003815.1)	*C. psittaci*<br>MN<br>(SRR652461.1)
C. psittaci<br>6BC<br>(CP002549.1)	*C. psittaci*<br>6BC<br>(CP002549.1)	*C. trachomatis*<br>A/HAR-13<br>(GCA_000012125.1)	*C. psittaci*<br>M56<br>(CP003814.1)	*C. psittaci*<br>6BC<br>(CP002549.1)	*C. psittaci*<br>NJ1<br>(CP003798.1; CP003816.1)	*C. psittaci*<br>NJ1<br>(SRR652467.1)
C. serpentis<br>H15-1957-10C<br>(LT993738.1)	*C. serpentis*<br>H15-1957-10C<br>(LT993738.1)		*C. psittaci*<br>MN<br>(CP003815.1)	*C. serpentis*<br>H15-1957-10C<br>(LT993738.1)	*C. psittaci*<br>VS225<br>(CP003793.1; CP003817.1)	*C. psittaci*<br>VS225<br>(SRR652453.1)
C. suis<br>SWA-14<br>(LT860207.1)	*C. suis*<br>SWA-14<br>(LT860207.1)		*C. psittaci*<br>NJ1<br>(CP003816.1)	*C. suis*<br>SWA-14<br>(LT860207.1)	*C. psittaci*<br>WC<br>(CP003796.1; CP003818.1)	*C. psittaci*<br>WC<br>(SRR652464.1)

C. trachomatis<br>A/HAR-13<br>(CP000051.1)	*C. trachomatis*<br>A/HAR-13<br>(CP000051.1)		*C. psittaci*<br>VS225<br>(CP003817.1)	*C. trachomatis*<br>D/UW-3/CX<br>(AE001273.1)	*C. psittaci*<br>WS/RT/E30<br>(CP003794.1; CP003819.1)	*C. psittaci*<br>WS/RT/E30<br>(CP003794.1)
Cand. C. corallus<br>G3/2742-324<br>(NWQK01000000.1)	*Cand.* C. corallus<br>G3/2742-324<br>(NWQK01000000.1)		*C. psittaci*<br>WC<br>(CP003818.1)	*Cand.* C. corallus<br>G3/2742-324<br>(NWQK01000000.1)		
Cand. C. ibidis<br>10-1398/6<br>(APJW01000000.1)	*Cand.* C. ibidis<br>10-1398/6<br>(APJW01000000.1)		*C. psittaci*<br>WS/RT/E30<br>(CP003819.1)	*Cand.* C. ibidis<br>10-1398/6<br>(APJW01000000.1)		
Cand. C. sanzinia<br>G4/2742-308<br>(CP014639.1)	*Cand.* C. sanzinia<br>G4/2742-308<br>(CP014639.1)		*C. serpentis*<br>H15-1957-10C<br>(LT993739.1)	*Cand.* C. sanzinia<br>G4/2742-308<br>(CP014639.1)		
	Simkania negevensis<br>Z<br>(FR872582.1)		*C. suis*<br>SWA-14<br>(LT860208.1)			
			C. trachomatis<br>A/HAR-13<br>(CP000052.1)			
			Cand. C. sanzinia<br>G4/2742-308<br>(CP014640.1)			
			Cand. C. corallus<br>G3/2742-324<br>(CM008979.1)			

Table S11. Strains used in this study and their GenBank accession numbers.
